# Oxygen limitation and tissue metabolic potential of the African fish *Barbus neumayeri*: roles of native habitat and acclimatization

**DOI:** 10.1186/1472-6785-11-2

**Published:** 2011-01-20

**Authors:** Mery L Martínez, Erin L Raynard, Bernard B Rees, Lauren J Chapman

**Affiliations:** 1 Department of Biology, Laurentian University, Sudbury, Ontario, P3E 2C6, Canada; 2 Department of Biological Sciences, University of New Orleans, New Orleans, Louisiana 70148, USA; 3 Department of Biology, McGill University, 1205 Avenue Docteur Penfield, Montréal, PQ, 3HA 1B1, Canada; 4 Wildlife Conservation Society, 185th Street and Southern Boulevard, Bronx, New York, 10460, USA

## Abstract

**Background:**

Oxygen availability in aquatic habitats is a major environmental factor influencing the ecology, behaviour, and physiology of fishes. This study evaluates the contribution of source population and hypoxic acclimatization of the African fish, *Barbus neumayeri*, in determining growth and tissue metabolic enzyme activities. Individuals were collected from two sites differing dramatically in concentration of dissolved oxygen (DO), Rwembaita Swamp (annual average DO 1.35 mgO_2 _L^-1^) and Inlet Stream West (annual average DO 5.58 mgO_2 _L^-1^) in Kibale National Park, Uganda, and reciprocally transplanted using a cage experiment in the field, allowing us to maintain individuals under natural conditions of oxygen, food availability, and flow. Fish were maintained under these conditions for four weeks and sampled for growth rate and the activities of phosphofructokinase (PFK), lactate dehydrogenase (LDH), citrate synthase (CS), and cytochrome c oxidase (CCO) in four tissues, liver, heart, brain, and skeletal muscle.

**Results:**

Acclimatization to the low DO site resulted in lower growth rates, lower activities of the aerobic enzyme CCO in heart, and higher activities of the glycolytic enzyme PFK in heart and skeletal muscle. The activity of LDH in liver tissue was correlated with site of origin, being higher in fish collected from a hypoxic habitat, regardless of acclimatization treatment.

**Conclusions:**

Our results suggest that the influence of site of origin and hypoxic acclimatization in determining enzyme activity differs among enzymes and tissues, but both factors contribute to higher glycolytic capacity and lower aerobic capacity in *B. neumayeri *under naturally-occurring conditions of oxygen limitation.

## Background

Because the concentration of dissolved oxygen (DO) in water is influenced by many factors, including temperature, depth, salinity, and eutrophication, organisms living in aquatic environments can be subjected to temporal and spatial variation in DO. Among fishes, low levels of DO, or hypoxia, is associated with impaired growth and reproduction [1-3]. If severe, hypoxia can lead to high rates of mortality in fish and other aquatic organisms depending upon their degree of hypoxia tolerance [4-6]. Aquatic hypoxia is a major environmental stressor that has become a global issue [7,8], and some predictions suggest that current global climate change will further exacerbate the problem [9,10]. Thus, it has become increasingly important to understand mechanisms that fish use in order to persist and survive under hypoxia.

Intraspecific variability in physiological traits plays a key role in the capacity of fish to adjust to low oxygen. Such variability can be attributed to genetic differentiation, phenotypic plasticity, or a combination of both. The time course of phenotypic responses to low oxygen varies from minutes or hours (e.g., acute responses) to days or weeks (development, acclimation, and acclimatization). Depending on the duration and intensity of hypoxia exposure, fish may display adjustments at behavioural, morphological, biochemical, and physiological levels [2,5,11-13]. Generally, during brief or moderate levels of hypoxia, fish attempt to maintain the same level of metabolic function observed under normal oxygen levels (normoxia); however, during prolonged or severe hypoxia, fish employ various mechanisms to enhance hypoxia tolerance. One of the major challenges to surviving hypoxia is balancing energy production and expenditure. Accordingly, under severe hypoxic stress, fish tend to reduce their metabolic demands, a response that is frequently coupled with changes in metabolic enzyme activities [14,18]. However, the degree and direction of enzyme adjustments depend upon the duration and severity of hypoxia [19-21], as well as the pathway, tissue, and species studied [2,22,23]. Moreover, evidence for adjustments in tissue metabolic potential (enzyme activities) in fish is almost exclusively drawn from laboratory acclimation studies.

In this study, we used a field experiment with the African fish *Barbus neumayeri *Fischer to study the role of acclimatization to low oxygen in determining growth and metabolic enzyme activities. *B. nemayeri *is a member of the cyprinid family, which is one of the most abundant and widespread freshwater families in Africa [24,25]. *B. neumayeri *is widely distributed in eastern Africa and inhabits a diversity of environments that display a great range of variation in DO, from chronically hypoxic swamps to well-oxygenated open rivers [1,25]. Previous studies have shown population specific variation in traits related to behaviour, respiration, morphology, and metabolism that may enhance hypoxia tolerance in populations from low DO habitats [1,20,26-28]. Here, we use reciprocal transplant acclimatization as our experimental design, which is not often used in physiological studies of fish. However, it is of great importance, since individuals are exposed to many other natural factors (predation, food availability, water flow among others) that can affect the overall capacity of organisms to respond adequately to the main factor under study, in this case hypoxia. We use this design to determine the influence of site of origin versus oxygen acclimatization treatment on growth and tissue enzyme activities. The enzyme activities measured were chosen to reflect the tissue capacities for anaerobic and aerobic carbohydrate metabolism.

## Results

Morphological variables of *B. neumayeri *used in this field transplant experiment are shown in Table 1. Fish were selected at the beginning of the experiment in a narrow size range (*L*_*S *_= 36 to 64 mm) to minimize size variation. Nonetheless, starting *M*_*T *_and *L*_*S *_were significantly lower for fish collected from Inlet Stream West (*M*_*T*_: *F*_1,77 _= 4.652, *P *= 0.034; *L*_*S*_: *F*_1,77 _= 4.998 *P *= 0.028), although condition (K) did not differ between collection sites. After the four week acclimatization period, *M*_*T*_, *L*_*S*_, and K did not differ for fish from different collection sites or exposed to different acclimatization treatments. Specific growth rate (G_S_), however, was significantly affected by acclimatization treatment, being lower for fish acclimatized to the low oxygen habitat (*F*_1,70 _= 5.346, *P *= 0.024, Table 1), as well as being significantly affected by cage assignment (*F*_9,70 _= 2.788, *P *= 0.008). The latter effect was for fish in cages holding fewer fish (lower density) to have higher G_S_. Other than G_S_, cage assignment was not a significant factor in the nested ANOVAs for any other response variable (morphological or enzymatic).

**Table 1:** **Morphological traits of the African cyprinid *Barbus neumayeri *collected from and acclimatized to field sites differing in dissolved oxygen content**.

	Acclimatization Site			
	**Inlet Stream West (normoxia)**		**Rwembaita Swamp (hypoxia)**	
	
	**Site of Origin**			
	
**Parameters**	**Inlet Stream West**	**Rwembaita Swamp**	**Inlet Stream West**	**Rwembaita Swamp**

**Initial *M***_***T***_** (g)**^*a*^	2.26 ± 0.23 (21)	2.84 ± 0.16 (21)	2.56 ± 0.25 (21)	2.68 ± 0.12 (27)
**Initial *L***_***S***_** (mm)**^*a*^	49.1 ± 1.7 (21)	53.4 ± 1.0 (21)	51.8 ± 1.89 (21)	53.0 ± 0.9 (27)
**Initial *K***	1.79 ± 0.03 (21)	1.82 ± 0.03 (21)	1.71 ± 0.02 (21)	1.77 ± 0.03 (27)
**Final *M***_***T***_** (g)**	2.17 ± 0.21 (21)	2.56 ± 0.17 (20)	2.45 ± 0.25 (21)	2.50 ± 0.11 (25)
**Final *L***_***S***_** (mm)**	48.4 ± 1.7 (21)	52.1 ± 1.0 (21)	50.2 ± 1.99 (20)	51.4 ± 1.0 (24)
**Final *K***	1.79 ± 0.03 (21)	1.78 ± 0.05 (20)	1.79 ± 0.05 (20)	1.84 ± 0.04 (24)
***G***_***S***_** (% d**^**-1**^**)**^*b,c*^	-0.10 ± 0.02 (20)	-0.12 ± 0.01 (19)	-0.15 ± 0.02 (20)	-0.14 ± 0.01 (24)

*Barbus neumayeri *were collected from Inlet Stream West (DO = 5.4 mg O_2_ L^-1^) and Rwembaita Swamp (DO = 1.1 mg O_2_ L^-1^) and held under a reciprocal acclimatization treatment for four weeks. Total mass (*M*_*T*_), standard length (*L*_*S*_), condition factor (*K*) were measured before and after the acclimatization treatment and specific growth rate (*G*_*S*_) was determined for the four week experiment. Data are shown as mean ± *SEM *with sample size in parentheses.

^***a ***^Means were significantly different for fish from different sites of origin (*P *< 0.05).

^***b ***^Means were significantly different for fish held at different acclimatization sites (*P *< 0.05).

^***c ***^Means were significantly different among cages within acclimatization site (*P *< 0.05).

**Figure 1:**
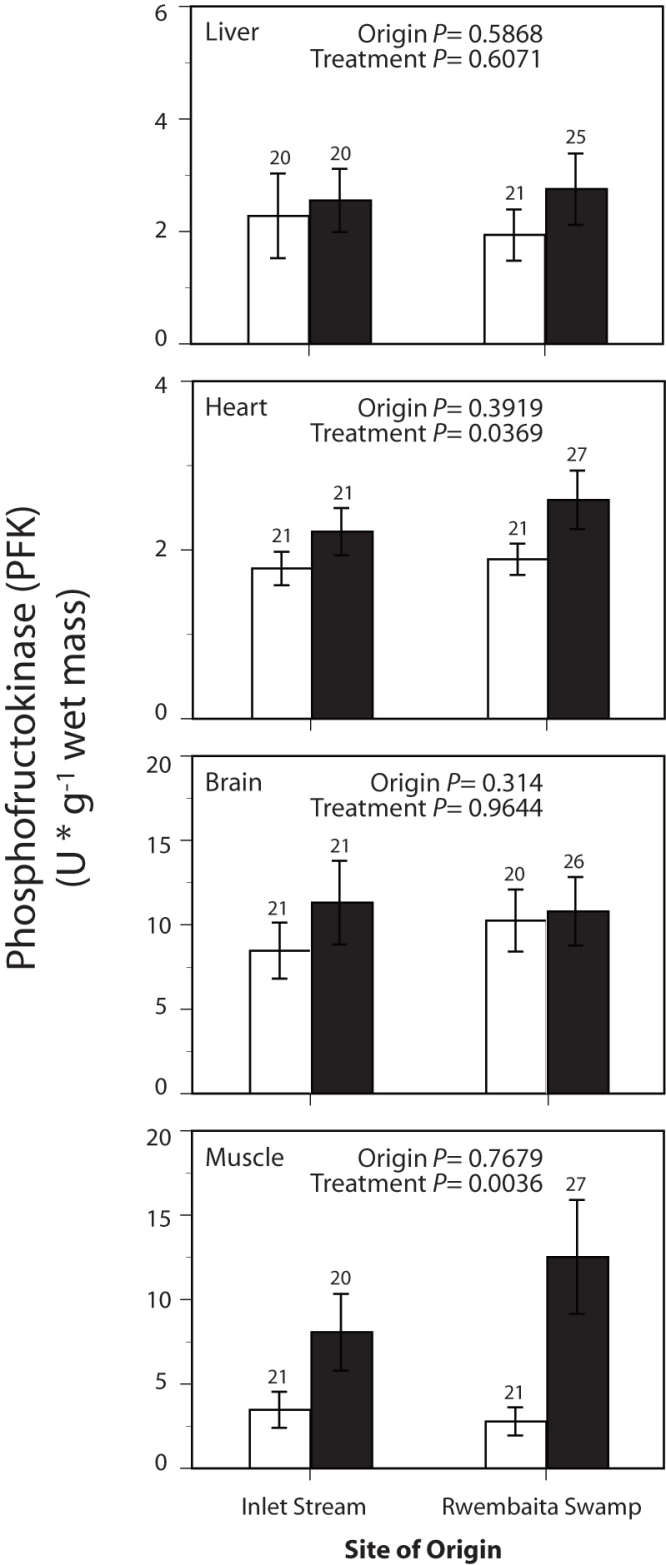
**Effects of collection site and acclimatization treatment on phosphofructokinase activities in tissues of the African cyprinid *Barbus neumayeri***. Samples were collected from Inlet Stream West or Rwembaita Swamp and acclimatized in the normoxic stream site (open bars) or the hypoxic swamp site (filled bars) for four weeks. *P *values are from two-way ANOVAs with site of origin and acclimatization treatment as main effects (see Methods). Error bars represent one *SEM *and sample sizes for each tissue are given above the bars.

**Figure 2:**
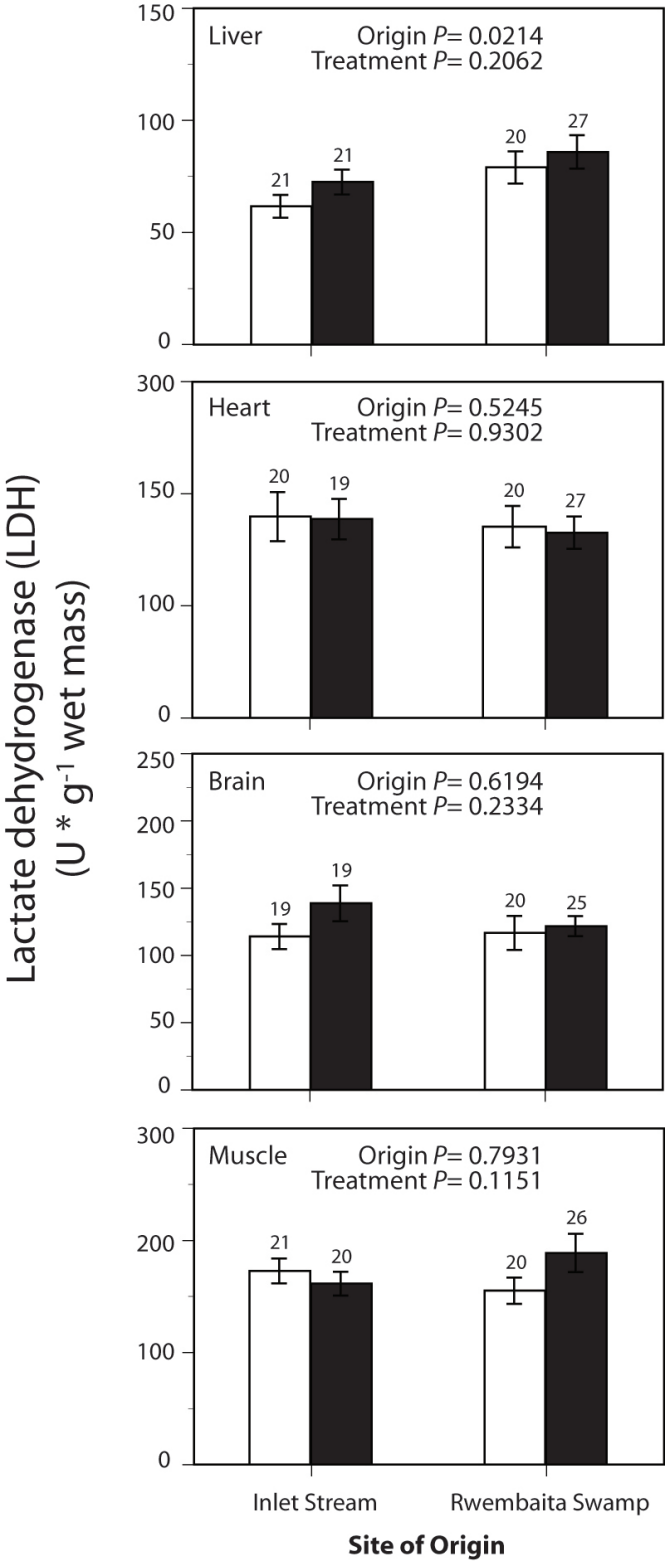
**Effects of collection site and acclimatization treatment on lactate dehydrogenase activities in tissues of the African cyprinid *Barbus neumayeri***. Samples were collected from Inlet Stream West or Rwembaita Swamp and acclimatized in the normoxic stream site (open bars) or the hypoxic swamp site (filled bars) for four weeks. For brain, mass-corrected LDH activities are shown. *P *values are from two-way ANOVAs with site of origin and acclimatization treatment as main effects (see Methods). Error bars represent one *SEM *and sample sizes for each tissue are given above the bars.

**Figure 3:**
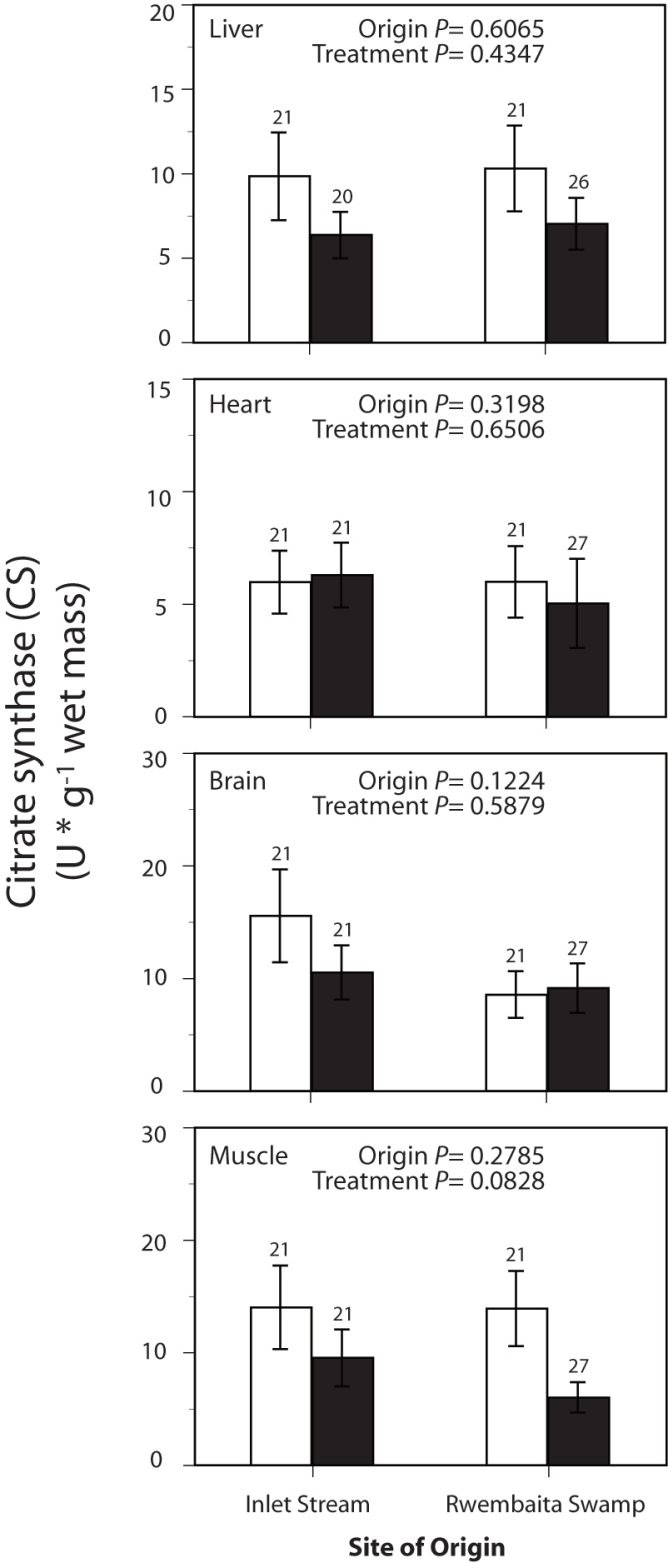
**Effects of collection site and acclimatization treatment on citrate synthase activities in tissues of the African cyprinid *Barbus neumayeri***. Samples were collected from Inlet Stream West or Rwembaita Swamp and acclimatized in the normoxic stream site (open bars) or the hypoxic swamp site (filled bars) for four weeks. *P *values are from two-way ANOVAs with site of origin and acclimatization treatment as main effects (see Methods). Error bars represent one *SEM *and sample sizes for each tissue are given above the bars.

**Figure 4:**
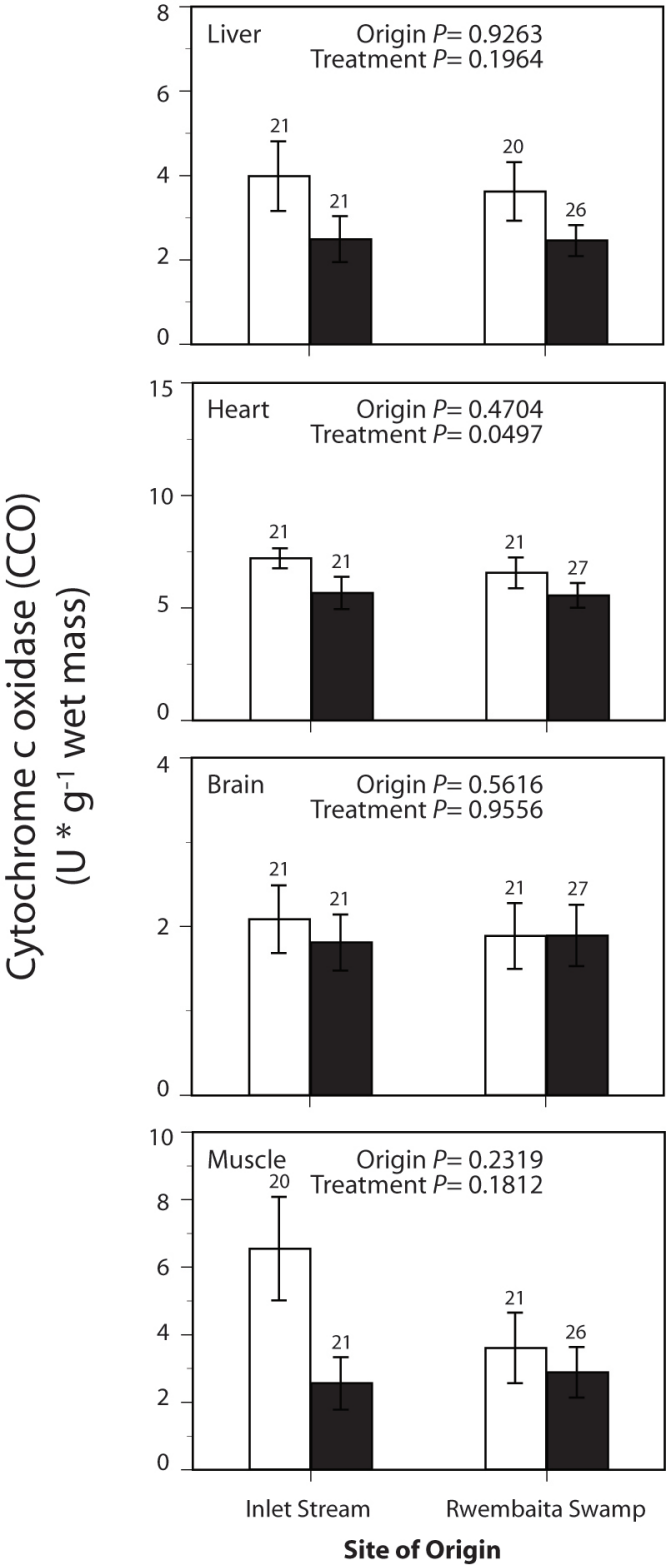
**Effects of collection site and acclimatization treatment on cytochrome c oxidase activities in tissues of the African cyprinid *Barbus neumayeri***. Samples were collected from Inlet Stream West or Rwembaita Swamp and acclimatized in the normoxic stream site (open bars) or the hypoxic swamp site (filled bars) for four weeks. *P *values are from two-way ANOVAs with site of origin and acclimatization treatment as main effects (see Methods). Error bars represent one *SEM *and sample sizes for each tissue are given above the bars.

Maximal enzyme activities representing tissue glycolytic capacity (PFK and LDH) and aerobic capacity (CS and CCO) are shown in Figure 1, Figure 2, Figure 3 and Figure 4. Exploratory data analysis indicated that body mass was positively related to brain LDH activity (*P *= 0.0048), and hence mass-corrected activities are shown for this enzyme only (Figure 2). In general, acclimatization site proved to be more important than collection site (origin) in determining the maximal activities of these enzymes. Acclimatization to the low oxygen habitat resulted in higher PFK activities in heart and skeletal muscle (*F*_1,76 _= 4.514, *P *= 0.037 and *F*_1,76 _= 9.035, *P *= 0.004, respectively, Figure 1). Conversely, fish acclimatized to the hypoxic swamp had lower heart CCO activity (*F*_1,77 _= 3.974, *P *= 0.049, Figure 4). Trends toward higher glycolytic enzyme activities and lower aerobic enzyme activities were observed in other tissues (e.g., muscle CS, Figure 3), but these were not statistically significant largely due to the high variation in enzyme activity determinations. Site of origin was significantly related to liver LDH activity: fish collected from the hypoxic swamp habitat had higher activities than fish from the normoxic stream habitat (*F*_1,76 _= 5.534, *P *= 0.021, Figure 2). The interaction between site of origin and acclimatization site was not significant for any tissue enzyme activity.

Because acclimatization treatment significantly affected G_S _(Table 1) and certain tissue enzyme activities (Figures 1 and Figure 4), correlation analysis was used to assess the relationship between G_S _and enzyme activity. Of the three enzyme activities that were significantly influenced by acclimatization treatment, only heart PFK was correlated with G_S _(r = -0.29, *P *= 0.009). This negative relationship, while statistically significant, explained less than 10% of the variation in heart PFK. The other two enzyme activities affected by acclimatization treatment, muscle PFK and heart CCO, were not related to G_S _(*P *> 0.05).

## Discussion

Oxygen availability in aquatic habitats is a major ecological factor influencing the distribution of fishes. The capacity of fish to survive, and even thrive, under conditions of low oxygen varies markedly among and within species, with more tolerant individuals being able to exploit low oxygen habitats. Fishes of East Africa provide outstanding examples of inter- and intraspecific variation in hypoxia tolerance. Previous research on *B. neumayeri *has shown that populations inhabiting low oxygen swamps differ in respiratory behaviour [26], gill morphology [27-29], and tissue metabolic capacity [20], all of which presumably enhance the hypoxia tolerance of individuals from these swamp populations. Herein, we evaluate the influence of source population and acclimatization to low oxygen in determining growth and metabolic potential of *B. neumayeri *from habitats differing in oxygen availability.

Among the various morphological and enzymatic measurements reported, only liver LDH activity was significantly related to site of origin, with individuals collected from hypoxic habitats displaying greater activities. This observation is consistent with our earlier observation that *B. neumayeri *from Rwembaita Swamp had higher liver LDH activity than fish from the normoxic Njuguta River [20]. In the current study, the effect of site of origin was observed after four weeks of field acclimatization to different oxygen habitats, whereas in the previous study, the effect of collection site was measured immediately after collection as well as after long term laboratory acclimation to normoxia. Although LDH plays a critical role in anaerobic metabolism, it is also involved in the process of gluconeogenesis by catalyzing the conversion of lactate into pyruvate, which can then be used for glucose production. Consequently, the role of higher LDH activity in livers from fish collected in the hypoxic Rwembaita Swamp may be in the clearance of blood lactate and the provision of glucose for metabolism by extra-hepatic tissues such as heart and brain, which are important in the maintenance of an organism's homeostasis [30-32].

The observation of differences between the Rwembaita Swamp and the inlet stream population populations of *B. neumayeri *in liver LDH activities after field acclimatization (this study) and laboratory acclimation [20], suggest a potential genetic component. This is supported by recent results using nuclear markers (CK and TPI-A) that show significant genetic divergence between Rwembaita Swamp and Inlet Stream West populations of *B. neumayeri *(Harniman unpublished data). Although we found strong evidence for plastic effects in some enzymatic traits, local adaptation to divergent oxygen regimes may also contribute to the observed trait variation as has been observed in other studies of fishes experiencing divergent selective environments [33-35].

One theoretical expectation arising from local adaptation is that organisms adapted to their native environment should perform "better" than organisms transplanted from their native environment and acclimatized to a novel habitat. This expectation would be experimentally supported in our study by a significant origin by acclimatization interaction with respect to potential fitness correlates such as growth. In this study, growth rates were negative in all groups, regardless of site of origin or acclimatization treatment, ranging from - 0.1% to - 0.15% body mass per day. It can be argued that the negative growth observed in this study was due to the artificial conditions of the experimental enclosures (e.g., increasing stress, reducing food availability); however, for *B. neumayeri *population within this system, it is quite natural for fish to become trapped in small pools and experience mass loss during the dry season [1,27]. In a mark and recapture study of the foraging ecology of *B. neumayeri *during the summer season, Schaack and Chapman [36] reported negative growth rates similar to those reported here.

Interestingly, mass loss was greater, on average, for fish acclimatized to the hypoxic Rwembaita Swamp compared to those in the normoxic Inlet Stream West, irrespective of site of origin. The restrictive effect of low oxygen on growth is consistent with numerous laboratory and field studies demonstrating reduced growth in fish under hypoxia [38]. Low growth under hypoxia in other species has been attributed to reduced rates of ingestion or assimilation efficiency or both [16,39,40]. Previous work on *B. neumayeri *demonstrated a trade-off between respiratory and trophic morphology, such that relatively larger gilled swamp fish spent more time handling food items than smaller gilled stream fish [27]. In the current experiment, this trade-off did not result in lower growth in fish originating from hypoxic swamps when compared to fish from the normoxic stream in either acclimatization treatment. This result indicates that under these experimental conditions, this putative trade-off was without adverse effects on growth. It is possible that other, unmeasured fitness tradeoffs contribute to the maintenance of differences between these populations in LDH, haematocrit, and morphological traits such as gill size [27].

Acclimatization to differing oxygen levels also affected tissue levels of metabolic enzymes. In particular, PFK was greater in heart and skeletal muscle from individuals acclimatized to the low oxygen swamp site, while CCO was lower in heart after hypoxic acclimatization. Phosphofructokinase is an important regulatory enzyme of glycolysis, and changes in PFK activity are likely to impact the flux through the glycolytic pathway. Higher PKF activities after hypoxic acclimatization, therefore, could represent an increased capacity for glycolytic ATP production. Cytochrome c oxidase, on the other hand, catalyzes the final step in mitochondrial electron transport to oxygen, and its rate is important in determining rates of aerobic ATP production. The significant effect of acclimatization on heart CCO was mirrored by a non-significant trend toward lower muscle citrate synthase (P = 0.08), a mitochondrial enzyme catalyzing the first step of the Kreb's cycle. The overall effect of acclimatization to low oxygen, therefore, was an increase in glycolytic capacity (heart and muscle PFK) and a trend towards decreased aerobic capacity (heart CCO and muscle CS). Results from laboratory acclimation of fish to hypoxia have yielded similar, if sometimes mixed results [reviewed by 2]. Nevertheless, the current data are an important field validation of predictions based upon energetic considerations and laboratory studies.

The present study employed a reciprocal transplant experimental design that is not commonly used in physiological studies. There are certainly challenges to this field experiment, for example the escape of fish from one replicate cage and the sporadic loss of individuals from other cages, likely due to predators that could enter the cages through the open tops (e.g., birds, snakes, and spiders). Another potential limitation of the study is that it was carried out at specific locations of known DO level, and to extrapolate our results more broadly it would be necessary to replicate this study in other systems having divergent oxygen concentrations. In addition, it is possible that the cage restriction itself altered the response of the fish to the acclimatization treatment. However, free swimming *B. neumayeri *collected at this and other Kibale field sites had negative growth rates [36], condition factor [41], and tissue LDH activities [20] similar to those measured in fish after the 4-week acclimatization treatment. Despite these concerns, our results suggest that reciprocal transplant experiments provide a powerful tool in uncovering physiological responses to naturally occurring ecological stresses.

## Conclusions

In this study, the relative contribution of native habitat and acclimatization to low oxygen were evaluated in the African cyprinid fish, *Barbus neumayeri *in a reciprocal transplant field experiment. The biological end-points measured included variables related to fitness (growth rate) and tissue metabolic potential (enzyme activities). Pronounced gradients in environmental oxygen availability affect multiple features of the respiratory behaviour, morphology, and physiology of *B. neumayeri*. For some traits, e.g, gill morphology [27] and liver LDH activities [[20]; present study], intraspecific variation persists after animals are removed from the field or transplanted to field sites differing in oxygen, arguing for a role of local adaption, whereas other traits, e.g., heart PFK and CCO activities, are more plastic and influenced by recent exposure to differing oxygen regimes. Thus, acclimatization of tissue metabolic potential may be an important response to the spatial mosaic of dissolved oxygen availability in this system. Together, local adaptation and acclimatization appear to be operating to enhance this species' capacity to live in divergent oxygen environments.

## Methods

### Study sites

*Barbus neumayeri*, a widely distributed cyprinid in Africa [25], were collected from two sites with very different DO levels but relatively similar in other abiotic parameters [28,36] in the Rwembaita Swamp system of Kibale National Park in western Uganda (0°13'-0°'41'N and 30°19'-30°32'E). Rwembaita Swamp is one of the largest swamps in Kibale National Park. It is 6.5 km in length and feeds into the Njuguta River, which is a tributary of Mpanga River that drains into the Lake George basin [28]. The hypoxic site, located in the central area of the Rwembaita Swamp, displayed a monthly mean DO concentration of 1.35 ± 0.18 mg L^-1 ^over a 2-year period [28]. The water current is extremely slow in the swamp, when compared with the numerous streams that drain into the swamp, and current is non-detectable in some areas during the dry season. One of the several streams that feed into the Rwembaita Swamp was selected as the normoxic site, the Inlet Stream West. This stream has low-flowing water current throughout much of the year, with periods of higher flows during flooding season. The monthly mean DO over 2 years was 5.58 ± 0.16 mg L^-1 ^for Inlet Stream West [28]. Despite the DO differences in both sampling sites, almost no diel variation in temperature and DO exist at each of the sites used here [29].

### Experimental design

For this study, individuals were captured at the two study sites using minnow traps, and a reciprocal transplant experiment was designed to determine the roles of collection habitat and acclimatization to alternative dissolved oxygen environments. Six cages were placed in Inlet Stream West, and six cages were placed in Rwembaita Swamp. Cylindrical cages (100 cm height × 80 cm diameter) were made from black plastic meshing (2 mm mesh size), except for the top, which was left open. The meshing allowed for free flow of water and food. Each cage was placed in water approximately 30 to 50 cm in depth and held in place with four poles implanted into the sediment or tied to adjacent trees.

Fish were haphazardly allocated into two densities, low and high, to explore the influence of density on growth rate and enzyme activity. Three low density cages and three high density cages were placed in each of the two sites. In each of the low density cages three swamp fish and three stream fish were placed; while in the high density cages, six swamp fish and six stream fish were placed. Before placing the fish into the cages, each individual was measured for standard length (*L*_*S *_± 1 mm) and total body mass (*M*_*T *_± 0.1 g), and tattooed under the skin with ink from a fine-gauge needle for individual identification [36]. The reciprocal acclimatization treatment lasted four weeks.

### Tissue sampling

Fish were netted and euthanized with an overdose of buffered MS-222 (1 g MS-222 and 4 g NaHCO_3 _L^-1^), and measured again for *M*_*T *_and *L*_*S*_, after which fish were individually wrapped in tin foil and frozen in a dry shipper. Fish were maintained in the dry shipper during transport to the University of Florida and then shipped to Laurentian University on dry ice, and finally stored at - 80°C until analyzed.

Tissues were dissected on ice in the following order: liver, heart, brain, and white skeletal muscle. Tissues were weighed and homogenized in 50 mM imidazole buffer (pH 7.5), 30 mM NaF, 2 mM ethylenediamine tetraacetic acid, 5 mM β-mercaptoethanol, 0.2 mM phenylmethyl-sulphonylflouride, 50 μg ml^-1 ^soybean trypsin inhibitor buffer, and 0.2% Triton 100X [20]. The liver, brain, and muscle tissue samples were homogenized in nine volumes of buffer; while heart tissue samples were homogenized in 49 volumes of buffer. Homogenates were made using a Polytron homogenizer (Polytron 1200C, Kinematica, Switzerland) for three 20 s periods. The samples were maintained on ice during and between the periods of homogenization. All homogenates were centrifuged at 2400 *g *for 15 min at 4°C, and supernatant solutions were kept on ice until enzyme activities were assayed.

### Enzyme assays

Immediately prior to enzyme assays, liver, brain, and muscle supernatants were diluted in homogenization buffer to get an overall 100-fold dilution in relation to the starting tissue mass. For each enzyme in each tissue, the concentrations of substrates, cofactors, and linking enzymes were optimized to give maximal activities. Reaction conditions for the determination of PFK and LDH enzyme activities were modified from [42]. The reactions for the aerobic enzymes, CS and CCO, were modified from [43]. The optimized reaction conditions were as follows:

*Phosphofructokinase *(PFK; E.C. 2.7.1.11.): 50 mM imidazole (pH 7.5), 20 mM KCl, 10 mM MgCl_2_, 0.17 mM NADH, 1 mM ATP (liver, heart and brain) or 2 mM ATP (muscle), 2 mM AMP (liver, heart and brain) or 4 mM AMP (muscle), 10 i.u. ml^-1 ^glycerol-3-phosphate dehydrogenase, 29 i.u. ml-^1 ^triosephosphate isomerase and 1 i.u. ml^-1 ^aldolase (liver, heart and muscle) or 2 i.u. ml^-1 ^aldolase (brain). Reactions were initiated with the addition of 5 mM fructose 6-phosphate.

*Lactate dehydrogenase *(LDH; E.C. 1.1.1.27.): 50 mM imidazole (pH 7.0), 0.17 mM NADH. Reactions were initiated by the addition of 1 mM pyruvate.

*Citrate synthase *(CS; E.C. 4.1.3.7.): 50 mM Tris (pH 8.0), 0.1 mM DTNB, 0.2 mM acetyl coenzyme A. Reactions were initiated by the addition of 0.3 mM oxaloacetate.

*Cytochrome c oxidase *(CCO; E.C. 1.9.3.1): 61.5 mM KH_2_PO_4 _(pH 7.0), 38.5 mM K_2_HPO_4_, 0.07 mM cytochrome c reduced. Reactions were run against a 70 μmol•L^-1 ^control of cytochrome c oxidized with 0.33% K-ferricyanide.

Maximal enzyme activities were measured in duplicate in a Varian spectrophotometer (Cary 100 Bio Varian Scientific Inc. Palo Alto CA, USA) with a circulating refrigerated water bath. Activities were measured at 25 ± 1°C. All enzyme levels were measured within 5 hours of the tissue homogenization to minimize decay of enzyme activity. LDH and PFK activities were measured at 340 nm to follow the disappearance of NADH. CCO activity was measured at 550 nm to follow the oxidation of reduced cytochrome c, and CS activity was measured at 412 nm to detect the transfer of coenzyme A to 5, 5'-dithiobis-2-nitrobenzoic acid (DTNB). The extinction coefficients for NADH, cytochrome C, and DNTB were respectively, 6.22, 19.1 and 13.6 cm^-1 ^μmol^-1^. Enzyme activities were expressed in international units (μmol substrate transformed to product min^-1^) g^-1 ^tissue mass. Biochemicals and chemicals were purchased from Sigma-Aldrich (St. Louis, U.S.A.), Boehringer Mannheim Co. (Montreal, Canada) and Fisher Scientific Co. (Montreal, Canada).

### Calculations and statistical analyses

Fulton's condition factor (K) was calculated as:

$$\text{K}=\left(M_{T}*L_{S}{}^{-\text{3}}\right)*\text{1}0^{\text{5}}$$

where *M*_*T *_is in g and *L*_*s *_is in mm.

Specific growth rate (G_S_) in % mass change per day was calculated as:

$${\text{G}}_{\text{S}}=\text{1}00*\left({\text{e}}^{\text{G}}-\text{1}\right)$$

where G = (ln final *M*_*T *_- ln initial *M*_*T *_)(t)^-1^, and *t *is the total number of days of the acclimatization period [40,44].

All response variables (morphology, growth, and enzyme activities) were compared against the normal distribution using one-sample Kolmogorov-Smirnov tests, and variables that were not normally distributed were log transformed. Two-way analyses of variance (ANOVA) were used to evaluate the effects of collection site (stream vs. swamp) and acclimatization treatment (site of cage; normoxic stream vs. hypoxic swamp), as well as their interaction, on response variables. Collection site and acclimatization treatment are fixed factors in this analysis because they do not represent a random sample of all possible field sites, but rather sites of known differences in dissolved oxygen level [45]. Although the density of fish was varied as part of the experimental design, all of the fish from one low density cage escaped and fish from other cages were occasionally lost due to predation, thereby altering the fish density. Therefore, density was not included as a fixed factor. Instead, cage assignment was nested within acclimatization treatment to account for differences in fish density and random factors associated with cage placement (e.g., microhabitat, food availability, competitors, and predators). Body mass was significantly correlated with brain LDH activity, and *M*_*T *_was included in the final model as a covariate for this enzyme only. Log transformation failed to normalize CS activities from heart, brain, and muscle, and consequently non-parametric Kruskall-Wallis tests were used to detect differences between collection site and acclimatization treatment. All statistical analyses were performed with Systat 10.2 and *P *< 0.05 indicated statistical significance.

## List of abbreviations

(AMP): Adenosine monophosphate; (ATP): adenosine triphosphate; (CS): citrate synthase; (CK): creatine kinase; (CCO): Cytochrome c oxidase; (DO): dissolved oxygen; (DTNB): 5, 5'-dithiobis-2-nitrobenzoic acid; (MS-222): ethyl 3-aminobenzoate methane-sulfonate salt; (K): Fulton's condition factor; (LDH): lactate dehydrogenase; (NADH): nicotine adenine dinucleotide; (PFK): Phosphofructokinase; (*G*_*S*_): specific growth rate; (*L*_*S*_): standard length; (*t*): time (number of days); (*M*_*T*_): total body mass; (TPI A): triose phosphate isomerise.

## Authors' contributions

MLM, BBR and LJC conceived the study and participated in its design and coordination, data analyses and manuscript preparation. ELR performed the lab work. LJC collected the samples. MLM, BBR, LJC performed the statistical analyses. All the authors contributed equally to the work in discussing research strategy and data interpretation. All authors read and approved the final manuscript.
